# Effectiveness of epinephrine auto‐injector skill training for school nurses

**DOI:** 10.1002/jgf2.282

**Published:** 2019-10-08

**Authors:** Yuko Shiraishi, Yukiko Ishikawa, Yoshihiko Shiraishi, Masami Matsumura

**Affiliations:** ^1^ Division of General Internal Medicine Jichi Medical University Hospital Shimotsuke Japan; ^2^ Oki‐Dozen Hospital Nishinoshima Japan

## Abstract

We investigated the effectiveness of epinephrine auto‐injector (EpiPen®) skill training for Yogo teachers in the community medicine. The auto‐injector skill training might be effective for establishing a caring system for anaphylaxis at schools in the context of community medicine.

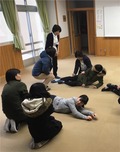

A fatal accident caused by anaphylactic shock after eating a school lunch in Japan was reported by the news media in 2012. An eighteen percent increase in food allergies in schoolchildren in the United States was reported from 1997 to 2007 by the Centers for Disease Control and Prevention.^1^ A prompt caring system for treatment of anaphylaxis caused by food allergy in schoolchildren should be established as soon as possible.^2^ In order to promote such a system, providing appropriate education to school nurses is of major importance.^3^ This prompted us to hold epinephrine auto‐injector (EpiPen^®^) skill training for *Yogo* teachers (*Yogo *teachers are participants in a specific system in Japan. They educate students about health‐related matters and provide health care to them.) in the community medicine of Dozen area. Dozen area consists of three islands. It is located in the Japan Sea and 50 kilometers off‐shore Shimane Peninsula. The Oki‐Dozen Hospital is the only public hospital in Dozen area. Since the doctors of the hospital are primary care physicians, they provide the emergency medical care for allergic problems and prescribe EpiPen^®^ for schoolchildren.

After the fatal accident by anaphylactic shock at a school in Japan in 2012, the *Yogo* teachers working group in Oki‐gun requested physicians to hold EpiPen^®^ training sessions. A training course was held in 2013 and was made known to all schools in Oki‐gun. Twenty‐one *Yogo* teachers from 21 schools (11 elementary schools, 6 junior high schools, and 4 high schools) took part in the auto‐injector skill training. The average age of teachers was 37.4 years (aged from 22 to 59), and average working period as a *Yogo* teacher was 15.4 years (the period from 8 months to 37 years). Nobody had got EpiPen^®^ training nor experience of EpiPen^®^ injection. First, the physician (YS) lectured about the necessity, indication, and methods of injection, and then provided EpiPen^®^ injection skill training. All participants trained to use the EpiPen^®^ practice device on a partner as instructed by the physician (Figure 1). We conducted questionnaires pretraining and posttraining as a part of managing our training. The institutional review board of Jichi Medical University approved this study. The response rate was 71.4% (15/21). Pretraining, every participant knew “EpiPen^®^”; however, seven participants (7/15, 46.7%) answered that they were not able to use EpiPen^®^. Posttraining, no participants answered that they could not use EpiPen^®^. We also asked participants about their pre‐ and posttraining confidence in using EpiPen^®^ expressed by a numeric rating scale (0‐100) and analyzed by the paired *t* test. The mean ± standard deviation scale of pre‐ and posttraining confidence scales increased from 40.8 ± 27.1 to 81.7 ± 10.1 (*P* < .001). The second training course was held in 2017. Pretraining, only three schools had a manual for anaphylaxis. However, 17 of 21 schools had developed a manual for anaphylaxis in 2018. Eight months after the second training, one of the participants did inject EpiPen^®^ to a schoolchild who had an episode of anaphylactic shock after eating a school lunch and saved his life.

**Figure 1:**
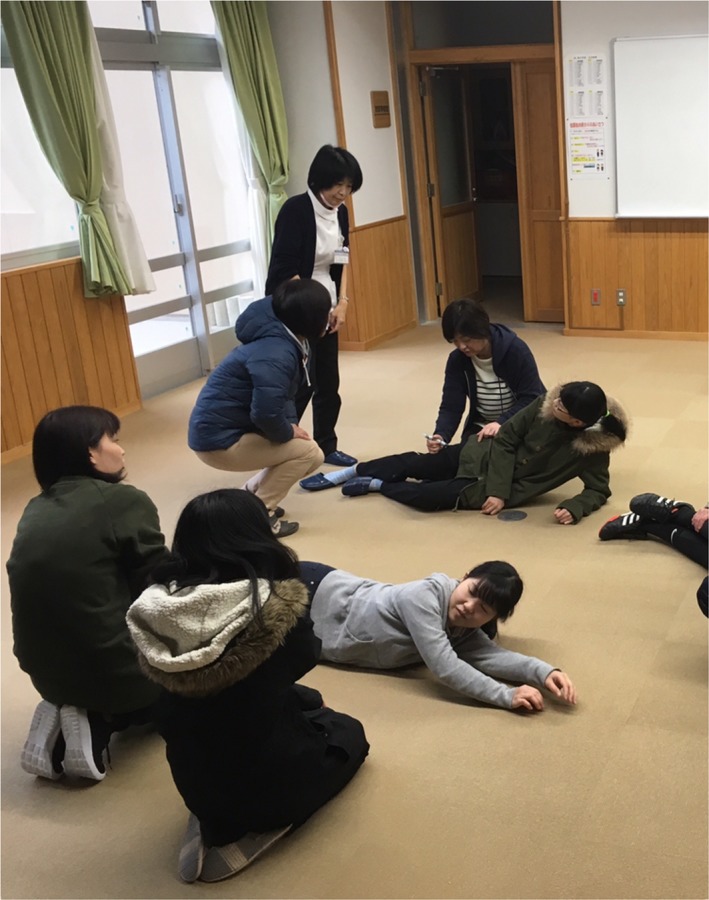
After the lecture, participants trained to use EpiPen^®^ practice device on a partner

These results indicated that the preparedness for anaphylaxis in school had been inadequate pretraining, while our program might motivate management for anaphylaxis and reduce anxiety regarding prompt EpiPen^®^ use. Effective collaboration between *Yogo* teachers and physicians was established through this activity, since all *Yogo* teachers in this area participated in the training. The achievement of providing training and the success of *Yogo* teachers' injection might be promoted by the close relationship between schools and a medical provider in the island community. The auto‐injector skill training might be effective for establishing a caring system for anaphylaxis at schools in the context of community medicine.

## CONFLICT OF INTEREST

The authors declare no conflict of interests for this article.

## PHOTOGRAPHIC CONSENT

Consents for publication of the photograph on Figure 1 were obtained from participants.
